# Ethanol extract of *Garcinia kola* seeds alleviates HGHFD/STZ-induced nonalcoholic fatty liver disease in diabetic rats by modulating oxidative stress, inflammation, and lipid accumulation

**DOI:** 10.22038/ijbms.2025.82786.17889

**Published:** 2025

**Authors:** Xiaojing Sun, Yanxiang Yuan, Xianhao Xin, Ping Sun, Yunqi Sun, Mi Xie, Yuefei Wang, Shan Huang, Bin Li

**Affiliations:** 1 Department of Pharmaceutical Engineering and Pharmaceutical Chemistry, College of Chemical Engineering, Qingdao University of Science and Technology, Qingdao, 266042, China; 2 National Key Laboratory of Chinese Medicine Modernization, State Key Laboratory of Component-based Chinese Medicine, Tianjin Key Laboratory of TCM Chemistry and Analysis, Tianjin University of Traditional Chinese Medicine, Tianjin, 301617, China

**Keywords:** Garcinia kola, HepG2, Lipid accumulation, SREBP-1c, T2DM combined with - NAFLD

## Abstract

**Objective(s):**

To investigate the ameliorative effects of *Garcinia kola* ethanol extract (EGK) on type 2 diabetes mellitus (T2DM) combined with nonalcoholic fatty liver disease (NAFLD) and to explore its underlying mechanisms.

**Materials and Methods:**

*In vivo*, a T2DM rat model was established using HGHFD/STZ. *In vitro*, HepG2 cells were induced with FFA to create a model of lipid accumulation. Lipid accumulation (LA), oxidative stress (OS) levels, and inflammatory markers were measured using kit methods. Additionally, the expression of the SREBP-1c pathway was detected by immunohistochemistry and western blot (WB) to further understand the potential mechanism of EGK’s protective effect on diabetic liver injury.

**Results:**

*In vivo*, EGK significantly reduced blood glucose levels (*P*<0.01), restored body weight (*P*<0.01), and improved liver LA, OS, and inflammatory levels (*P*<0.01) in diabetic rats. Histopathological results indicated that EGK effectively ameliorated diabetes-induced liver injury. Immunohistochemistry and WB results revealed that EGK significantly down-regulated the expression of the SREBP-1c pathway (*P*<0.01). *In vitro*, EGK markedly improved lipid accumulation, oxidative stress, and inflammation levels in HepG2 cells (*P*<0.01). Immunofluorescence and WB results showed that EGK significantly reduced the expression of the SREBP-1c pathway (*P*<0.01).

**Conclusion:**

EGK alleviates T2DM combined with NAFLD by reducing lipid accumulation through the inhibition of oxidative stress, inflammatory responses, and the SREBP-1c signaling pathway.

## Introduction

Type 2 diabetes mellitus (T2DM) is a chronic metabolic disease with persistent hyperglycemia resulting from insulin resistance and is a significant global health problem. (1). Hyperglycemia can lead to numerous complications, including cardiovascular disease, diabetic nephropathy, diabetic retinopathy, etc., and can also lead to liver metabolic disorders, thereby inducing NAFLD (2). Currently, about one-quarter of adults globally are affected by NAFLD, with approximately 70% of individuals with T2DM developing NAFLD (3). Without effective treatment, patients with T2DM combined with NAFLD (T2DM-NAFLD) are at risk of further progressing to NASH, liver fibrosis, and cirrhosis (4, 5).

The liver plays a vital role in lipid metabolism (LM), serving as the central organ responsible for uptake, synthesis, catabolism, and oxidation of lipids (6). In diabetic patients, hepatic lipid metabolic disorder can lead to abnormal lipid accumulation, thereby resulting in steatosis and ultimately leading to the onset and progression of T2DM-NAFLD (7). SREBP-1c, as a crucial transcription factor in LM, regulates the synthesis of free fatty acids (FFA) and triglycerides (TG) by controlling the expression of enzymes such as fatty acid synthase (FAS), acetyl-CoA carboxylase (ACC), and stearoyl-CoA desaturase-1 (SCD-1), consequently playing a central role in hepatic lipid metabolism (8, 9). In T2DM, insulin resistance can abnormally activate SREBP-1c, leading to excessive accumulation of FFAs and TGs, which results in the overproduction of ROS and subsequently activates inflammatory signaling pathways, releasing large amounts of inflammatory cytokines and contributing to the onset and progression of NAFLD (10, 11).

Currently, the primary medications for treating T2DM-NAFLD include oral hypoglycemic agents, PPAR agonists, GLP-1R agonists, and SGLT2 inhibitors. These drugs effectively reduce blood glucose (BG) and lipid levels, hence contributing to the improvement of T2DM-NAFLD (12). However, these drugs are often accompanied by adverse reactions, including hypoglycemia and lactic acidosis (13). In contrast, traditional herbal medicines, noted for their multi-component, multi-target, and multi-pathway regulation with minimal toxicity, provide a comprehensive approach to treating T2DM-NAFLD (14).

*Garcinia kola* seeds have attracted considerable attention due to their rich nutritional content (15). Numerous studies have shown that *G. kola* possesses various pharmacological activities, including treating hypertension and bronchitis, anti-inflammatory, anti-oxidant, hypoglycemic, and hypolipidemic effects, and hepatoprotective actions (16). In previous studies, we isolated and identified 14 compounds from the ethanol extract of *G. kola* (EGK), including kolaflavanone, tocopherol, xanthone, garcinoic acid, kaempferol-3-O-β-D-glucoside, 1-O-coumaroyl glucose, p-hydroxybenzaldehyde, 3-indolecarboxaldehyde, chrysochlamic acid, δ-(E)-deoxy-amplexichromanol, GB-1: 3’,5,5’,7,7’-pentahydroxy-2,2’-bis(4-hydroxyphenyl)-[3,8’-bichromane]-4,4’-dione, GB-2: 2’-(3,4-dihydroxyphenyl)-3’,5,5’,7,7’-pentahydroxy-2-(4-hydroxyphenyl)-[3,8’-bichromane]-4,4’-dione, GB-1a: 5,5’,7,7’-tetrahydroxy-2,2’-bis(4-hydroxyphenyl)-[3,8’-bichromane]-4,4’-dione, and kolaflavanone, with GB-1 and GB-2 being the major components of EGK (17). Previous studies have shown that *Garcinia kola* improves BG levels in T2DM db/db mice and alleviates insulin resistance and hepatic lipid accumulation in high-fat diet-fed mice (18, 19). Our previous research has also demonstrated that EGK can alleviate oxidative stress (OS) and inflammatory responses in HGHFD/STZ-induced diabetic mellitus (DM) rats and inhibit glomerular mesangial apoptosis (20). Additionally, it has been reported that GB-1 and GB-2 improve lipid metabolism in HepG2 cells by regulating PPARα (21). However, studies on EGK for T2DM-NAFLD have not yet been reported.

Therefore, this study established a rat model of T2DM-NAFLD using HGHFD/STZ and a lipid accumulation model by inducing HepG2 with FFA. The therapeutic effects of EGK on T2DM-NAFLD were investigated both *in vivo *and *in vitro*, and its potential mechanisms of action were explored.

## Materials and Methods

### Preparation of EGK extraction

EGK (ethanol extract of *Garcinia kola* seeds) was prepared following the method in our previous study (20). 

### HPLC (High-performance liquid chromatography) analysis of EGK

The chromatographic method used in this study was consistent with that described in our previous work, ensuring identical conditions and parameters (17).

### Experimental animals

Ninety male Sprague-Dawley (SD) rats, aged 6 weeks and weighing 200 ± 20 g, were obtained from the Qingdao Institute for Food and Drug Control (Qingdao, China; approval number: SYXK (Lu) 2022 0611). The rats were housed in an animal facility with a temperature of 22–24 ℃, 40%–60% relative humidity, a 12 hr light/dark cycle, and were provided with *ad libitum *access to food and water for 7 days (20). All animal experiments were conducted with the approval of the Institutional Animal Care and Use Committee (approval number: QKD-2024-21).

### Induction of T2DM rats and study design

Ten rats were assigned to the control group (CON) and given a standard diet, while the others were provided with a high-sugar (HG), high-fat diet (HFD) (60% standard diet, 20% sucrose, 10% lard, 9% egg yolk powder, and 1% cholesterol). After 8 weeks of HGHFD feeding, diabetes was induced by intraperitoneal injection of freshly prepared streptozotocin (STZ) at a dose of 35 mg/kg, dissolved in 0.1 M citrate buffer (pH 4.5). After 72 hr, blood was collected from the rats via the tail vein to measure fasting blood glucose (FBG). Rats with FBG levels of ≥11.1 mmol/l were classified as diabetic rats (n=50) and were chosen for further experiments.

The diabetic rats were randomly assigned to five groups (n = 10): the diabetic model group (MC); the metformin-treated group (Met, 0.25 g/kg); the low-dose EGK group (L-EGK, 0.107 g/kg); the medium-dose EGK group (M-EGK, 0.214 g/kg); and the high-dose EGK group (H-EGK, 0.428 g/kg). The CON group was maintained on a standard diet during the treatment period, while the EGK treatment and MC groups continued on the HGHFD. The rats were administered the respective treatments by gavage, with the CON and MC groups receiving an equivalent volume of normal saline for 8 weeks.

Throughout the study, FBG levels and BW were assessed weekly. Following treatment, the rats were euthanized, and blood was collected from the abdominal aorta. The serum was separated by centrifugation, and liver tissue samples were rinsed with physiological saline and stored at -80 ℃ for subsequent experiments.

### Biochemical analysis

Commercially available assay kits were used to quantify AST and ALT levels in serum and liver tissue.(22) Assay kits were used to measure TG, TC, HDL-C, LDL-C, SOD, GSH-Px, CAT, and MDA levels in rat liver tissue and HepG2 cells (23, 24). ELISA kits were used to determine TNF-α, IL-1β, IL-6, and CRP levels in liver tissue and HepG2 cells (25).

### Liver histopathological evaluation

Rat liver tissues were fixed in 4% paraformaldehyde, followed by dehydration with graded ethanol, paraffin embedding, and sectioning into 4 μm slices. For pathological examination, sections were stained with hematoxylin-eosin (H&E) and Masson’s trichrome. After fixation, liver sections were embedded in OCT compound, sectioned into 8 μm slices, and stained with Oil Red O. Stained sections were examined and imaged under a light microscope. Cells were stained with Oil Red O using the same procedure as for tissue samples.

### Liver immunohistochemical staining

After deparaffinization and rehydration of the liver tissue sections, antigen retrieval was conducted using a retrieval buffer. To inhibit endogenous peroxidase, sections were incubated in 3% H_2_O_2_ for 25 min. Overnight incubation at 4 ℃ with anti-SREBP-1c primary antibody (1:200) was performed on the sections. Following PBS washes, a one hour incubation with a secondary antibody (1:100) was performed on the sections. Diaminobenzidine (DAB) was used to visualize the antigen-antibody complexes, followed by hematoxylin counterstaining (26). The sections were examined with a light microscope, and images were taken. ImageJ was used to analyze and quantify the positive staining areas in the captured images.

### Cell culture and cell viability

HepG2 cells (human hepatocellular carcinoma) were cultured in DMEM supplemented with 10% FBS at 37 ℃. The cells were obtained from Wuhan Procell Life Science & Technology Co., Ltd. The MTT assay was used to assess cell viability. HepG2 cells (5×10^4^ cells/ml) were plated in 96-well plates and incubated for 12 hr. Following incubation, the cells were exposed to various concentrations of FFA (0.0625, 0.125, 0.25, 0.5, and 1 mM) for 48 hr or to different concentrations of EGK (50, 100, 200, 400, and 800 µg/ml) for 24 hr. 50 µl of MTT (2.5 mg/ml) was added to each well, followed by a 4 hr incubation. After dissolving formazan crystals in 150 µl of DMSO, absorbance was measured at 490 nm using a microplate reader.

### Immunofluorescence microscopy

After EGK incubation, the cells were fixed in 4% paraformaldehyde for 30 minutes and then permeabilized with 0.1% Triton X-100. Overnight incubation at 4 ℃ with the SREBP-1c antibody (1:2000) was performed on the cells. Cells were washed with PBS after incubation, followed by a one hour incubation with a secondary antibody (1:100). Nuclei staining with DAPI (1 µg/ml, 10 min) was followed by fluorescence microscopy imaging and ImageJ analysis.

### Western blot analysis

Proteins were isolated from liver tissue and HepG2 cells using RIPA lysis buffer supplemented with 1% PMSF protease inhibitor. Protein levels were quantified using a BCA assay kit. SDS-PAGE was used to separate target proteins, which were then transferred to a PVDF membrane. After blocking with 5% non-fat milk for 2 hr, the membrane was incubated with primary antibodies for SREBP-1c, ACC, FAS, and SCD-1 at room temperature for 1.5 hr. Afterward, the membrane was incubated with an HRP-conjugated secondary antibody. Enhanced chemiluminescence (ECL) was used for protein detection, and ImageJ was used to quantify band intensities.

### Statistical analysis

Statistical analysis of all data was conducted using IBM SPSS Statistics 26.0 software (SPSS Inc, USA) and GraphPad Prism 8.0 software (GraphPad Software, Inc., San Diego, CA, USA). The data are expressed as mean ± standard deviation (SD) and followed by the normal distribution test. One-way ANOVA was used to compare experimental results, followed by individual *post hoc* Tukey’s test as appropriate. Statistical significance was defined as *P*<0.05. Each experiment was independently repeated three times.

## Results

### HPLC Chromatograms of EGK

**Figure 1:**
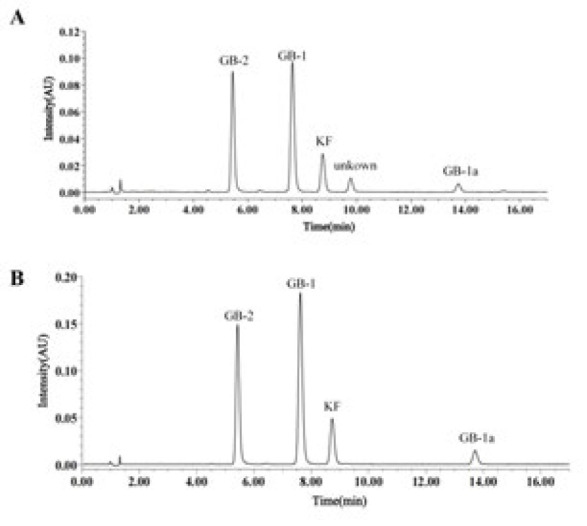
Description of components in *Garcinia kola* seed extract

GB-2, GB-1, KF, and GB-1a were identified as the main components of EGK through HPLC. The representative chromatograms of EGK analyzed by HPLC (Figure 1A) and the standards (Figure 1B) are shown in Figure 1.

### Effect of EGK on liver injury and lipid accumulation in diabetic rats

**Figure 2:**
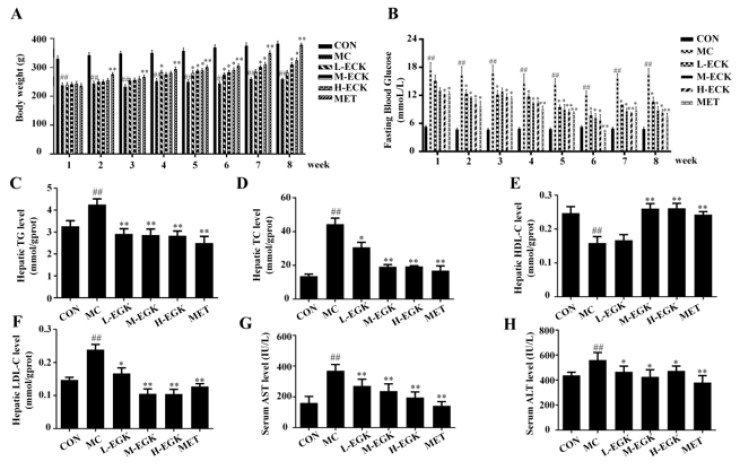
Effects of EGK on T2DM-related parameters

As shown in Figure 2, the body weight of rats in the MC group was markedly decreased compared to the CON group, and after EGK treatment, the body weight significantly elevated progressively with increasing doses. Additionally, the FBG level in the MC group increased, while EGK treatment significantly reduced it. Furthermore, the levels of TG, TC, and LDL-C in the liver tissues of the MC group were markedly increased (*P*<0.01), while HDL-C levels were pronouncedly reduced, and EGK treatment could reverse these changes. Liver injury markers ALT and AST increased in the MC group, indicating liver damage, but EGK could reverse these changes, suggesting its protective effects against liver injury. The data show that HGHFD/STZ induces lipid accumulation and liver injury in rats, and EGK treatment alleviates this condition.

### Effect of EGK on oxidative stress (OS) and inflammatory factors in the kidney of diabetic rats

**Figure 3:**
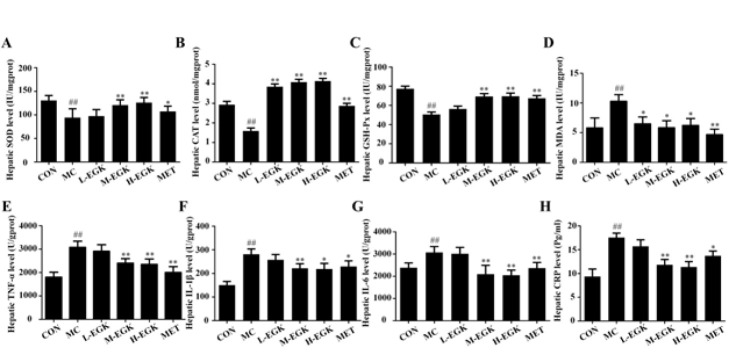
Effects of EGK on hepatic oxidative stress and inflammatory marker levels in T2DM rats

Figure 3 illustrates the changes in OS and inflammation in T2DM-NAFLD. As shown in Figures 3A-C, the levels of hepatic SOD, CAT, and GSH-Px in the MC group were significantly reduced. After 8 weeks of treatment with EGK, these enzyme levels in the liver of diabetic rats were significantly increased, with H-EGK showing the most significant effect (*P*<0.05). Figure 3D shows that hepatic MDA levels were significantly increased in the MC group, but treatment with metformin and H-EGK significantly reversed this increase (*P*<0.01). The above results suggest that EGK can significantly improve oxidative stress imbalance in the liver of diabetic rats. As shown in Figures 3E-H, the hepatic TNF-α, IL-1β, IL-6, and CRP levels were significantly elevated in the MC group. However, after 8 weeks of treatment, M-EGK and H-EGK significantly reduced the levels of these inflammatory factors in diabetic rats (*P*<0.01 or *P*<0.05).

### Effect of EGK on liver histopathology in diabetic rats

**Figure 4:**
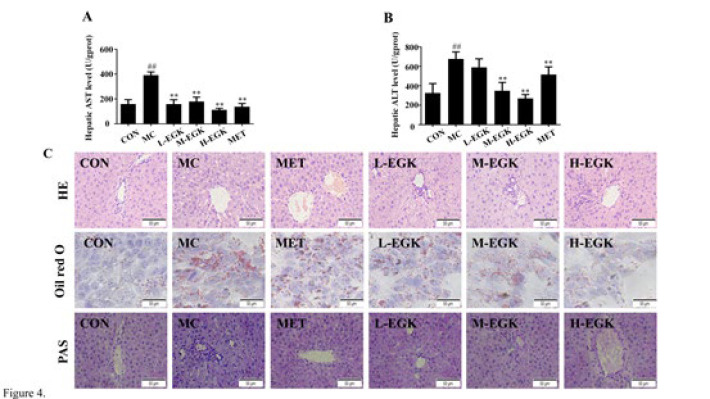
Effects of EGK on hepatic histopathological alterations T2DM rats

As shown in Figures 4A-B, the levels of AST and ALT in liver tissue align with the serum results, suggesting that EGK has a significant protective effect on the liver. H&E staining results showed no notable pathological changes in the CON group, while the MC group had severe hepatic ballooning degeneration, disrupted sinusoidal structure, disordered hepatocyte arrangement, and extensive lipid vacuoles and inflammatory cells. Oil Red O and PAS staining showed significant lipid droplet and glycogen accumulation in the MC group. In the M-EGK and H-EGK groups, there was a significant reduction in fat vacuoles, sinusoidal lesions, size and number of lipid droplets, and glycogen accumulation (Figure 4C). In summary, EGK reduced pathological liver injury, lipid degeneration, and hepatic glycogen accumulation in diabetic rats induced by HGHFD/STZ.

### Effect of EGK on gene expression of lipid metabolism in the liver of diabetic rats

**Figure 5:**
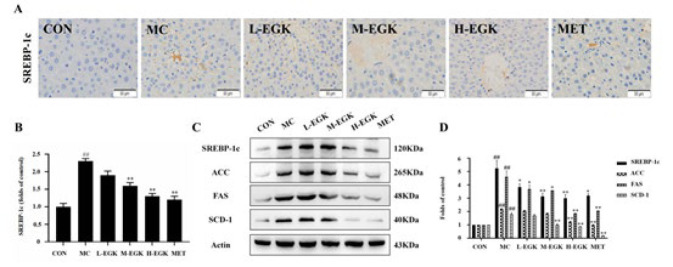
Effect of EGK on SREBP-1c pathway expression in the liver of T2DM rats

Immunohistochemical staining showed that the area of the SREBP-1c positive region was significantly increased in the MC group, which was significantly reduced following EGK and Met treatments (*P*<0.01), suggesting EGK inhibits its overexpression to lower lipid synthesis (Figures 5A-B). Western blot (WB) analysis in diabetic rats showed elevated SREBP-1c, ACC, FAS, and SCD-1 protein levels in the MC group, which EGK treatment decreased (Figures 5C-D), indicating EGK reduces liver lipid accumulation by suppressing these proteins.

### Effects of EGK on lipid accumulation in HepG2 cells

**Figure 6:**
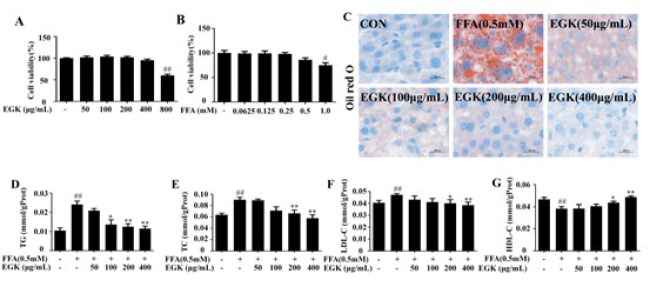
Effects of EGK on lipid accumulation and cellular damage in FFA-induced HepG2 cells

As shown in Figures 6A and B, neither the 48 hr treatment with 0.5 mM FFA nor the 24 hr treatment with EGK (50–400 µg/ml) had a significant inhibitory effect on the viability of HepG2 cells. The Oil Red O staining results indicate that FFA significantly induced steatosis in HepG2 cells, while EGK reduced lipid droplet deposition in a dose-dependent manner (Figure 6C). After FFA induction, the intracellular TG, TC, and LDL-C levels significantly increased, while HDL-C levels decreased significantly. However, under different concentrations of EGK treatment, these indicators were partially restored (Figures 6D-G). These findings suggest that EGK can inhibit FFA-induced fat deposition in HepG2 cells.

### Effects of EGK on the oxidative stress and inflammatory response in HepG2 cells

**Figure 7:**
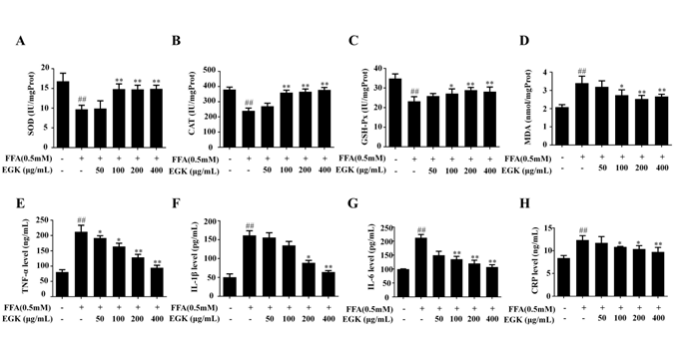
Effects of EGK on the levels of oxidative stress indicators and inflammatory markers in FFA-induced HepG2 cells

The results showed that after FFA induction, the activities of SOD, CAT, and GSH-Px in HepG2 cells were significantly reduced, while MDA content increased. However, EGK treatment reversed this situation (Figures 7A-D). Under FFA stimulation, the expression of pro-inflammatory factors TNF-α, IL-1β, IL-6, and CRP in HepG2 cells increased. After EGK intervention, this situation was significantly improved (Figures 7E-H). This indicates that EGK significantly inhibits oxidative stress and inflammatory response in HepG2 cells stimulated by FFA. 

### Effect of EGK on the FFA-induced SREBP-1c pathway in HepG2 cells

**Figure 8:**
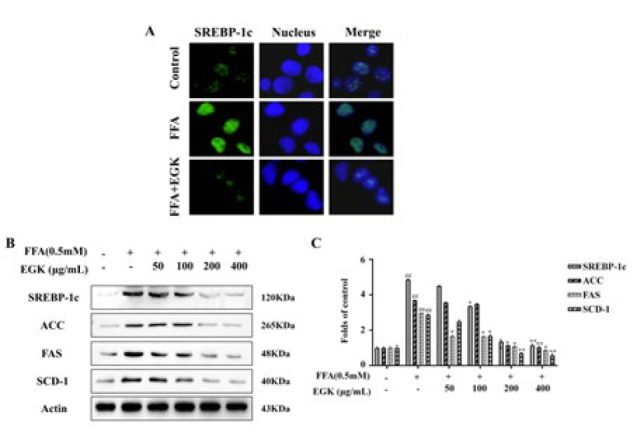
EGK could regulate the expression of lipid-related proteins in FFA-induced HepG2 cells

Figure 8 shows the expression of genes involved in lipogenesis and fatty acid oxidation in HepG2 cells. Immunofluorescence staining demonstrated that the expression of SREBP-1c in the FFA group was significantly increased, while EGK treatment reversed the expression (Figure 8A). Additionally, WB results indicated significantly increased SREBP-1c, ACC, FAS, and SCD-1 expression in the FFA group, which decreased following EGK treatment (*P*<0.05 or *P*<0.01). These results suggest that EGK ameliorates lipid accumulation in FFA-induced HepG2 cells by regulating the SREBP-1c pathway.

## Discussion

In recent decades, NAFLD has become a global public health concern, especially the liver disease induced by T2DM (27). In T2DM-NAFLD, the liver, as the primary target organ, plays a key role in glucose regulation and lipid homeostasis (28). T2DM, with metabolic dysregulation, promotes hepatic steatosis, which subsequently leads to various pathological developments of the liver.(29) Currently, the drugs used in the treatment of NAFLD and diabetes-induced liver diseases are mainly hypoglycemic drugs and liver protection drugs (30, 31). However, these medications often exhibit limited efficacy and are associated with severe side effects (32). Thus, significant attention has been focused on the development of safer and more effective alternative treatments derived from natural products.(33) *Garcinia kola* seeds, a traditional African medicine, contain biflavonoids and garcinoic acid derivatives (15). Through the HPLC analysis, we found that GB-1, GB-2, GB1a, and KF are the main components of the *Garcinia kola*. Studies have shown that in addition to the main components like GB-1 and GB-2, other compounds such as tocopherol and kolaviron also exhibit significant hypoglycemic, hypolipidemic, anti-oxidant, and anti-inflammatory effects (34). Although previous studies have shown that EGK has hypoglycemic and liver preservation effects in diabetic rats, there is currently no research on the therapeutic effects and mechanisms of EGK in T2DM-NAFLD.

In this investigation, we utilized HGHFD/STZ-induced diabetes mellitus (DM) rats to mimic the physiological conditions of T2DM-NAFLD patients. FFA-treated HepG2 cells were used to establish an *in vitro *fatty liver model. In the state of insulin resistance induced by T2DM, insulin fails to suppress glucose production and instead significantly elevates lipid levels, ultimately resulting in hyperglycemia and hypertriglyceridemia. The results showed that BG levels in DM rats were significantly elevated, and TG, TC, LDL-C, ALT, and AST levels were markedly increased in both DM rats and HepG2 cells. More importantly, histopathological analysis demonstrated significant structural alterations in the liver of the MC group rats, aligning with previous findings. Remarkably, these indicators were significantly improved following EGK treatment, and the pathological liver damage showed substantial recovery. Therefore, our findings indicate that EGK may ameliorate lipid accumulation and exert hepatoprotective effects by reducing body weight (BG), lipid, and liver enzyme levels in DM rats.

Oxidative stress (OS) is a crucial pathogenesis mechanism in T2DM-NAFLD (35). Abnormal lipid accumulation in patients disrupts the OS balance, which further exacerbates damage to the organism (36). As the main organ responsible for detoxification and oxidative stress regulation, the liver is highly sensitive to alterations in OS levels *in vivo* (37). OS occurs in an imbalance between oxidation and anti-oxidation, typically caused by increased free radical generation and reduced activity of anti-oxidant enzymes (37). Key anti-oxidant enzymes like SOD, CAT, and GSH-Px neutralize free radicals and are commonly used as biomarkers of oxidative stress levels (38). Under T2DM conditions, persistent high glucose, high fatty acids, and insulin resistance lead to the accumulation of reactive ROS within cells (39). Excessive ROS damage macromolecules, resulting in the generation of lipid peroxidation products like malondialdehyde (MDA), which indirectly reflect the extent of organ oxidative damage. Multiple studies have demonstrated that anti-oxidant enzyme activity is suppressed while MDA levels increase in DM rats and patients with NAFLD.(40–42) Our results align with previous studies, showing significant increases in GSH-Px, SOD, and CAT levels and a significant decrease in MDA levels in the liver tissue of DM rats and FFA-induced HepG2 cells after EGK treatment. These findings imply that EGK may alleviate T2DM-NAFLD by inhibiting OS.

OS can initiate a sequence of cascades comprising the activation of inflammation signaling, which exacerbates inflammatory damage to the liver (43). Previous research has demonstrated that EGK down-regulates NF-κB activation, which is a critical regulatory factor for inflammation and immune homeostasis (44). Under oxidative stress stimulation, NF-κB induces the gene expression of pro-inflammatory cytokines. CRP is an important marker for evaluating liver inflammation. Excessive TNF-α, IL-1β, and IL-6 release induces CRP production in the liver, thereby exacerbating hepatic inflammatory responses (45). In this study, our findings revealed a significant reduction in TNF-α, IL-1β, and IL-6 levels in the livers of MC group rats after EGK treatment, accompanied by a decrease in CRP synthesis. Similar results were observed in FFA-induced HepG2 cells. These results suggest that EGK significantly ameliorates liver inflammation in HGHFD/STZ-induced T2DM-NAFLD rats.

Lipid accumulation is an important stage in the development of T2DM-NAFLD (46). Inflammatory responses, oxidative stress, increased TG synthesis, and lipid accumulation lead to hepatic lipid deposition in diabetic rats, accelerating the progression of T2DM-NAFLD (47). Previous studies have indicated that inhibiting lipogenesis and promoting fatty acid oxidation are essential methods for reducing hepatic lipid accumulation (48). It has been reported that increased hepatic lipogenesis and reduced fatty acid oxidation are closely linked to the development of T2DM-NAFLD (49). SREBP-1c is a critical transcription factor that regulates the expression of multiple genes associated with hepatic lipogenesis, including SCD-1, ACC, and FAS (50). By regulating the expression of these genes, SREBP-1c directly affects fatty acid synthesis, TG synthesis, and lipid accumulation, serving a pivotal function in regulating lipid balance and metabolic homeostasis (51). Among these, SCD-1 catalyzes the conversion of saturated to monounsaturated fatty acids and regulates fatty acid synthesis as a rate-limiting enzyme in fatty acid metabolism (52). Previous research has demonstrated a positive relationship between SCD-1 expression levels and hepatic fat content. Its overactivation promotes TG synthesis and lipid accumulation, leading to hepatic steatosis (53). Moreover, ACC functions as a rate-limiting enzyme in the fatty acid synthesis pathway, facilitating the conversion of acetyl-CoA to malonyl-CoA, a crucial intermediate in the process of fatty acid synthesis.(54) Studies have shown that increased ACC activity promotes long-chain fatty acid synthesis and reduces fatty acid oxidation, further exacerbating hepatic steatosis and lipid deposition within hepatocytes, contributing to the development of NAFLD (55). FAS catalyzes the elongation of fatty acids, converting malonyl-CoA and acetyl-CoA into long-chain fatty acids (56). Overexpression of FAS in NAFLD has been documented to increase fatty acid synthesis and contribute to TG formation, resulting in lipid accumulation and hepatic steatosis (57). Interestingly, our study found that EGK reduces TG synthesis by inhibiting the expression of SREBP-1c, SCD-1, ACC, and FAS proteins while simultaneously enhancing fatty acid oxidation. This mechanism significantly reduces lipid accumulation in HGHFD/STZ DM rats and FFA-induced HepG2 cells, thereby effectively inhibiting the progression of T2DM-NAFLD.

In parts (A and B), GB-2, GB-1, KF, and GB-1a. GB-1: 3’,5,5’,7,7’-pentahydroxy-2,2’-bis(4-hydroxyphenyl)-[3,8’-bichromane]-4,4’-dione; GB-2: 2’-(3,4-dihydroxyphenyl)-3’,5,5’,7,7’-pentahydroxy-2-(4-hydroxyphenyl)-[3,8’-bichromane]-4,4’-dione; GB-1a: 5,5’,7,7’-tetrahydroxy-2,2’-bis(4-hydroxyphenyl)-[3,8’-bichromane]-4,4’-dione; KF: Kolaflavanone

(A) Changes in body weights. (B) Glucose level. (C-F) The content of TG, TC, HDL-C, and LDL-C in the liver of treatment. (G-H) Content of AST and ALT in serum of treatment. Data are expressed as mean ± SD (n=10 in each group). #*P*<0.05 or ##*P*<0.01 vs CON; *P<0.05 or **P<0.01 vs MC.

T2DM: Type 2 diabetes mellitus; EGK: *Garcinia kola* ethanol; TG: Triglycerides; TC: Total cholesterol; HDL-C: High-density lipoprotein cholesterol; LDL-C: Low-density lipoprotein cholesterol; AST: Aspartate aminotransferase; ALT: Alanine aminotransferase

(A-D) Levels of oxidative stress markers (SOD, CAT, GSH-Px, and MDA) in the livers of all groups of rats. (E-H) Effect of EGK on liver TNF-α, IL-1β, IL-6, and CRP. Data are expressed as mean ± SD (n=10 in each group). #*P*<0.05 or ##P<0.01 vs CON; *P<0.05 or **P<0.01 vs MC.

EGK: Ethanol extract of *Garcinia kola* seeds; T2DM: Type 2 diabetes mellitus; SOD: Superoxide dismutase; GSH-Px: Glutathione peroxidase; MDA: Malondialdehyde; TNF-α: Tumor necrosis factor-α; IL-1β: Interleukin-1β; IL-6: Interleukin-6

(A) Hepatic ALT level. (B) Hepatic AST level. (C) Representative figures of histological abnormalities in diabetic liver tissues (400×), H&E staining was used for the analysis of histological abnormalities; Oil red O staining was used to detect liver fat deposition; PAS staining was used for the detection of glycogen (purple) in the liver tissues. Data are expressed as mean ± SD (n=10 in each group). #*P*<0.05 or ##*P*<0.01 vs CON; *P<0.05 or **P<0.01 vs MC.

EGK: Ethanol extract of *Garcinia kola* seeds; T2DM: Type 2 diabetes mellitus; ALT: Alanine aminotransferase; AST: Aspartate aminotransferase

(A) Images of SREBP-1c immunohistochemistry, scale bars = 50 µm. (B) Relative protein expressions of SREBP-1c. (C) Expression of lipid-related proteins of SREBP-1c, ACC, FAS, and SCD-1 by western blot. (D) The quantitative analysis of SREBP-1c, ACC, FAS, and SCD-1. #*P*<0.05 or ##*P*<0.01 vs CON; *P<0.05 or **P<0.01 vs MC.

EGK: Ethanol extract of *Garcinia kola* seeds; T2DM: Type 2 diabetes mellitus

(A-B) The viability of HepG2 cells following FFA and EGK intervention was tested through MTT assay. (C) Photomicrographs of Oil Red O staining of HepG2 cells. (D-G) The TG, TC, LDL-C, and HDL-C content in HepG2 cells. Data represented by means ± SD (n=6). #*P*<0.05 or ##*P*<0.01 vs -; *P<0.05 or **P<0.01 vs FFA group

EGK: Ethanol extract of *Garcinia kola* seeds; FFA: Free fatty acids; T2DM: Type 2 diabetes mellitus; TG: Triglycerides; TC: Total cholesterol; HDL-C: High-density lipoprotein cholesterol; LDL-C: Low-density lipoprotein cholesterol

(A) Intracellular SOD, (B) Intracellular CAT, (C) Intracellular GSH-Px, (D) Intracellular MDA, (E) Intracellular TNF-α, (F) Intracellular IL-1β, (G) Intracellular IL-6, (H) Intracellular CRP. Data represented by means ± SD (n=6). #*P*<0.05 or ##*P*<0.01 vs -; *P<0.05 or **P<0.01 vs FFA group

EGK: Ethanol extract of *Garcinia kola* seeds; FFA: Free fatty acids; SOD: Superoxide dismutase; GSH-Px: Glutathione peroxidase; MDA: Malondialdehyde; TNF-α: Tumor necrosis Factor-α; IL-1β: Interleukin-1β; IL-6: Interleukin-6

(A) Expression of SREBP-1c was displayed by immunofluorescence staining (400×). (B) Expression of lipid-related proteins of SREBP-1c, ACC, FAS, and SCD-1 by western blot. (C) Quantitative analysis of SREBP-1c, ACC, FAS, and SCD-1. #*P*<0.05 or ##*P*<0.01 vs -; *P<0.05 or **P<0.01 vs FFA group

EGK: Ethanol extract of *Garcinia kola* seeds; FFA: Free fatty acids; FAS: Fatty acid synthase; SCD-1: Stearoyl-CoA desaturase-1

## Conclusion

In summary, our study demonstrates that EGK administration can reduce serum ALT and AST levels in HGHFD/STZ-induced T2DM-NAFLD rats, decrease liver lipid accumulation, enhance liver anti-oxidant enzyme activity, and decrease the expression of liver lipogenesis genes, thereby exerting a therapeutic effect on T2DM-NAFLD.

## Declaration

We have not used any AI tools or technologies to prepare this manuscript.
